# Activated mast cells promote differentiation of B cells into effector cells

**DOI:** 10.1038/srep20531

**Published:** 2016-02-05

**Authors:** Anna-Karin E. Palm, Gianni Garcia-Faroldi, Marcus Lundberg, Gunnar Pejler, Sandra Kleinau

**Affiliations:** 1 Uppsala University, Department of Cell and Molecular Biology, Uppsala, Sweden; 2 Swedish University of Agricultural Sciences, Department of Anatomy, Physiology and Biochemistry, Uppsala, Sweden; 3 Uppsala University, Department of Medical Biochemistry and Microbiology, Uppsala, Sweden

## Abstract

Based on the known accumulation of mast cells (MCs) in B cell-dependent inflammatory diseases, including rheumatoid arthritis, we hypothesized that MCs directly modulate B cells. We show here that degranulated, and to a lesser extent naïve or IgE-sensitized, MCs activate both naïve and B cell receptor-activated B cells. This was shown by increased proliferation, blast formation, and expression of CD19, MHC class II and CD86 in the B cells. Further, MCs stimulated the secretion of IgM and IgG in IgM^+^ B cells, indicating that MCs can induce class-switch recombination in B cells. We also show that coculture of MCs with B cells promotes surface expression of L-selectin, a homing receptor, on the B cells. The effects of MCs on B cells were partly dependent on cell-cell contact and both follicular and marginal zone B cells could be activated by MCs. Our findings suggest that degranulated MCs support optimal activation of B cells, a finding that is in line with *in vivo* studies showing that MCs frequently degranulate in the context of B-cell driven pathologies such as arthritis. Together, our findings show that MCs have the capacity to differentiate B cells to effector cells.

Accumulating evidence has challenged the classical view of B cells depending on T cell help for full activation and maturation. Thus, it has been shown that a number of innate immune cells such as invariant natural killer T cells, dendritic cells, granulocytes and mast cells (MCs) can provide help for B lymphocytes to undergo somatic hypermutation and antibody class switch recombination (CSR) without the need for CD4^+^ T cells^1^,^2^,^3^,^4^,^5^,^6^,^7^,^8^. MCs are known to be involved both in innate and adaptive immune responses^9^ and are strategically located at the surfaces of the skin and mucosa of the respiratory, gastro-intestinal and genital tracts. B cells can also be found at mucosal surfaces, where they are required to produce mainly IgA and IL-10 in order to maintain a non-inflammatory milieu^10^,^11^,^12^,^13^. In this context, it has been shown that MCs can help B cells to switch to this phenotype^14^,^15^.

The classical connection between MCs and the adaptive immune response is represented by the ability of MCs to bind IgE, with MC activation by stimulation of the high affinity IgE receptor being a hallmark of allergic reactions^16^. Moreover, MCs are implicated to have a role in inflammatory diseases such as autoimmune arthritis^17^,^18^. Interestingly, both human patients with rheumatoid arthritis (RA) and mice subjected to the collagen-induced arthritis (CIA) RA model show increased numbers of MCs in the inflamed synovium^17^,^19^,^20^,^21^,^22^,^23^,^24^, suggesting that MCs contribute to this type of pathology. Indeed, there are several studies based on the use of MC-deficient animals that support a pathogenic role of MCs in various models of arthritis, both passively^25^ and actively^18^ induced. It is also well established that B cells have a non-redundant role in both CIA and RA^26^,^27^, with functions including the production of autoantibodies, secretion of cytokines and presentation of autoantigen.

Based on the well-documented accumulation of MCs in B cell-dependent inflammatory diseases, together with the reported functional impact of MCs in several models of B cell-driven inflammatory disease^28^, we here hypothesized that MCs might have the ability to directly modulate the activation and differentiation of B cells. To address this possibility, we cocultured naïve or B cell receptor (BCR)-activated B cells with MCs and analysed the effect of MCs on various parameters of B cell activation. We also evaluated the effects of MCs on follicular (FO) and marginal zone (MZ) B cells; two major B cell subsets with different immune functions: FO B cells participate in T-dependent immune responses that involve germinal centre reactions and production of high affinity IgG, whereas MZ B cells mainly produce the early wave of low-affinity IgM, and may switch to IgG independently of T cell stimulation^29^. In addition, MZ B cells are better antigen presenting cells and cytokine producers than FO B cells and may thus participate in the activation of naïve T cells^30^,^31^,^32^,^33^.

Indeed, we show that MCs can activate B cells, including both FO and MZ B cells, not only by inducing them to proliferate and differentiate into CD19^high^ blasts, but also by promoting B cell differentiation into an antigen-presenting phenotype with high surface expression of class II MHC (MHCII) and CD86. Moreover, IgM^+^ B cells cocultured with MCs underwent IgG CSR, further indicating a promotion of an effector B cell phenotype, and we also demonstrate that MCs promote the expression of the homing receptor L-selectin on B cells.

## Materials and Methods

### Ethics statement

All animal experiments were approved by the Uppsala animal research ethics committee (permit numbers C71/11, C72/11) or the Northern Stockholm’s animal research ethics committee (permit number N18/14). All experiments were carried out in accordance with the approved guidelines.

### Mice

DBA/1 mice of both sexes and at 12–26 weeks of age were used. They were originally obtained from Bommice, Bomholt Gaard Ltd (Ry, Denmark) and were bred and maintained at the animal facilities at either the Biomedical Centre, Uppsala University, Uppsala, Sweden or at the National Veterinary Institute, Uppsala, Sweden. The mice were fed rodent chow and water *ad libitum*, and were negative for routine-screened pathogens.

### Preparation of B cells

Spleens were collected post mortem and single-cell suspensions were prepared from individual mice by gently mashing the spleen through a stainless steel mesh. To lyse erythrocytes, ACK buffer (0.15 M NH_4_Cl (Merck KGaA, Darmstadt, Germany), 0.1 mM EDTA (Merck KGaA) and 1.0 M KHCO_3_ (Sigma-Aldrich, St. Louis, MO, USA)) was added followed by a wash in phosphate-buffered saline (PBS). The splenocytes were then enriched for B cells using magnetic-activated cell sorting (MACS) according to the manufacturer’s protocol (Miltenyi Biotec, Bergisch Gladbach, Germany). In brief, the splenocytes were labelled with anti-mouse CD43 MicroBeads (Miltenyi Biotec) at 4 °C for 30 min. Labelling was followed by washing and resuspension in MACS buffer (0.5% bovine serum albumin (BSA), 2mM EDTA in PBS). The cells were subsequently loaded onto an LS column (Miltenyi Biotec) in a magnetic field and the effluent, containing CD43^−^ naïve, mature B cells, was collected. The B cells were subsequently labelled with carboxyfluorescein succinimidyl ester (CFSE) using the Vybrant® CFDA SE Cell Tracer kit (Molecular Probes, Leiden, Netherlands) according to the manufacturer’s protocol. Briefly, 10 × 10^6^ cells were labelled for 7 minutes using 5 μM of the dye followed by 4 washes in Dulbecco’s modified Eagle’s medium (DMEM; National Veterinary Institute, Uppsala, Sweden) supplemented with 10 mM HEPES (Sigma-Aldrich), 100 U/ml penicillin (Sigma), 100 μg/ml streptomycin (Sigma), 2 mM glutamine (Sigma), 50 μM β-mercaptoethanol and 20% heat-inactivated foetal calf serum (FCS; Sigma), referred to as complete DMEM 20% FCS. Finally, the CFSE-labelled B cells were diluted in complete DMEM 10% FCS. The cells were kept on ice throughout the preparation. Cell counts and determination of viability were made using trypan blue (Gibco Island, NY, USA).

### Bone marrow-derived MCs (BMMCs)

BMMCs were differentiated by culturing bone marrow cells from mouse femurs and tibias in DMEM supplemented with 10% FCS (Invitrogen, Carlsbad, CA), 100 IU/ml of penicillin, 50 μg/ml of streptomycin, 2 mM L-glutamine, 30% WEHI-3B conditioned medium (as a source of IL-3) and further supplemented with 10 ng/ml recombinant mouse IL-3 (Peprotech, Rocky Hill, NJ, USA). Medium was replaced weekly, keeping cell suspensions at a concentration of 0.5 × 10^6^ cells/ml. After 3 weeks, BMMCs were antigen-activated to confirm full maturation before using them for experiments. Cells used in all experiments were cultured for 4–8 weeks. WEHI-3B-conditioned medium was produced by culturing WEHI-3B cells (0.5 × 10^6^ cells/ml) for 3 days and collecting the culture supernatant. The culture conditions and phenotype of BMMCs have been described previously^34^.

### Activation of BMMCs

The BMMCs were sensitized either with IgE anti-TNP (1 μg/ml) or IgE anti-DNP (0.1 μg/ml) overnight. For activation, the cells were washed, resuspended in Tyrode’s buffer (130 mM NaCl, 5 mM KCl, 1.4 mM CaCl_2_, 1 mM MgCl_2_, 5.6 mM glucose, 10 mM HEPES and 0.1% BSA, pH 7.4) and stimulated with TNP-ovalbumin (OVA) or DNP-human serum albumin (HSA), at optimal concentrations of 0.4 μg/ml or 0.5 μg/ml, respectively. For cocultures with B cells, activation of the BMMCs was done with the same amount of TNP-OVA or DNP-HSA added directly to the cultures.

### Coculture of B cells and BMMCs and flow cytometry analysis

B cells (1 × 10^6^ cells/ml) and BMMCs (1 × 10^6^ cells/ml; hereafter referred to as MCs) were cultured together in round-bottomed 96-well cell culture plates (Sarstedt, Nümbrecht, Germany). This B cell to MC ratio was found optimal by a titration study (data not shown). The B cells were either naïve or activated through cross-linking of the BCR using an anti-μ F(ab’)_2_ fragment specific antibody (anti-μ; Jackson ImmunoResearch Laboratories, Inc., West Grove, PA, USA) at 5 μg/ml. The MCs were either in resting state (non-sensitized) or IgE-sensitized in the absence or presence of TNP-OVA for activation via FcεRI as described above. After three days, 100 μl of culture supernatant was collected from each well, replicates pooled, and stored at −20 °C until analysis for IgM and IgG. The replicate cultures were then suspended in the residual volume, pooled and washed once in FACS buffer (PBS with 1% BSA fraction V (Merck KGaA). The cells were subsequently treated with Fc block (clone 24G2; BD Pharmingen, BD Biosciences, San Jose, CA, USA) and stained for flow cytometry using biotinylated anti-mouse MHCII I-A^q^ (clone KH116, BD Pharmingen, BD Biosciences) followed by APC-conjugated streptavidin (Biolegend, San Diego, CA, USA) and anti-mouse B220-APC-Cy7 (clone RA3-6B2; Biolegend), CD19-PE (clone 1D3, BD Bioscience), CD86-PE-Cy7 (clone GL1, Biolegend) and L-selectin-BV421 (clone MEL-14, BD Horizon, BD Biosciences). Both staining steps were performed for 30 minutes in the dark at 4 °C and were followed by two washes. Immediately before analysis, 5 μl of the viability stain 7-AAD (Biolegend) were added to each sample. Flow cytometry was performed using an LSRII flow cytometer (BD Biosciences) and the data were analysed using the FlowJo software 9.6.1 (Treestar, Ashland, OR, USA). B cells were gated as 7-AAD^−^B220^high^ (see Supplementary Fig. S1 online). MC-dependent B cell proliferation was assessed as the percentage of B cells in cocultures with lower CFSE intensity than B cells cultured alone. Blast formation was assessed as B cells being large in forward scatter. For the transwell experiments, HTS 96-transwell polystyrene plates with 0.4 μm pore size polycarbonate membranes (Costar Coring, Inc., Schiphol-Rijk, The Netherlands) were used. B cells were seeded in the lower well, and MCs were added to the top chamber of the transwell system. When B cells were cultured with conditioned medium from activated MCs, the MCs were activated as previously described for 3 days and their culture supernatant was added to the B-cell culture.

### Cytokine array

Secretion of cytokines was determined in 3-days conditioned medium from activated MCs, naïve or BCR-activated B cells and coculture of BCR-activated B cells plus activated MCs by using a RayBio mouse cytokine antibody array 3 (RayBiotech, Inc., Norcross, GA) according to the manufacturer’s instructions.

### ELISA

The measurement of total IgM and IgG antibodies in cell culture supernatants was done using ELISA. Ninety six-well MaxiSorp plates (NuncBrand Thermo Fischer Scientific, Roskilde, Denmark) were coated over night at 4 °C with polyclonal goat anti-mouse Ig and subsequently blocked with dry milk followed by addition of undiluted cell culture supernatant. For detection we used alkaline-phosphatase conjugated sheep anti-mouse IgM or IgG (Sigma-Aldrich, St. Louis, MO, USA). After each step the plates were washed in PBS with 0.05% Tween (Sigma-Aldrich) and finally developed using p-nitrophenyl phosphate substrate (Sigma-Aldrich) diluted in diethanoleamine buffer (1 mg/ml). The absorbance was measured at 405 nm using a spectrophotometer (VersaMax, Molecular devices, Sunnyvale, CA, USA).

### B cell sorting

For sorting of follicular (FO) and marginal zone (MZ) B cells, the splenocytes were first enriched for B cells using MACS magnetic cell separation as described above. The CD43^−^ B cells were then labelled with fluorochrome-conjugated anti-mouse CD1d-PE (clone 1B1; Biolegend) and anti-mouse CD23-FITC (clone B3B4; Biolegend) at 4 °C for 30 minutes. After washing, the cells were sorted into FO and MZ B cells using a BD FACSAriaIII and FACSDiva software v. 6.1.3 (BD Biosciences). FO B cells were defined as CD23^high^CD1d^low^, and MZ B cells as CD23^low^CD1d^high^. Contamination with CD5^+^ B cells or transitional B cells was considered negligible using this sorting protocol^35^ and the sorting purity was routinely around 90% (see Supplementary Fig. S2 online). For sorting of IgM^+^ B cells, CD43^−^ B cells were stained with biotinylated anti-IgM (clone R6-60.2, BD Pharmingen, BD Bioscience) followed by PE-conjugated streptavidin (Biolegend). IgM^+^ B cells were selected with a sorting purity of >98% (see Supplementary Fig. S3 online).

### Statistical analysis

To eliminate inter-experimental variation when analysing blast formation and surface expression of MHCII and CD86, a fold change was calculated to the internal control culture of B cells only. This was done separately for cultures with naïve and BCR-stimulated B cells to the respective control. For flow cytometry analysis of MZ and FO B cells in coculture with MCs, a fold change was calculated to the respective internal control for all parameters, both for naïve and BCR-stimulated B cells. Statistical analyses were performed using Prism 6.0d from GraphPad Software, Inc. (La Jolla, CA, USA). Statistical differences between ≥3 groups were determined using one-way ANOVA with a Fisher’s least significant difference test. When comparing the effect on MZ and FO B cells an un-paired two-tailed Student’s *t*-test was used. For analysing OD-values we used repeated measures 1-way ANOVA when comparing ≥3 groups, and a paired two-tailed Student’s *t*-test for comparing 2 groups. All results are presented as mean + SEM. P-values <0.05 were considered significant.

## Results

### MCs enhance proliferation and activation of naïve and BCR-activated B cells

**Figure 1:**
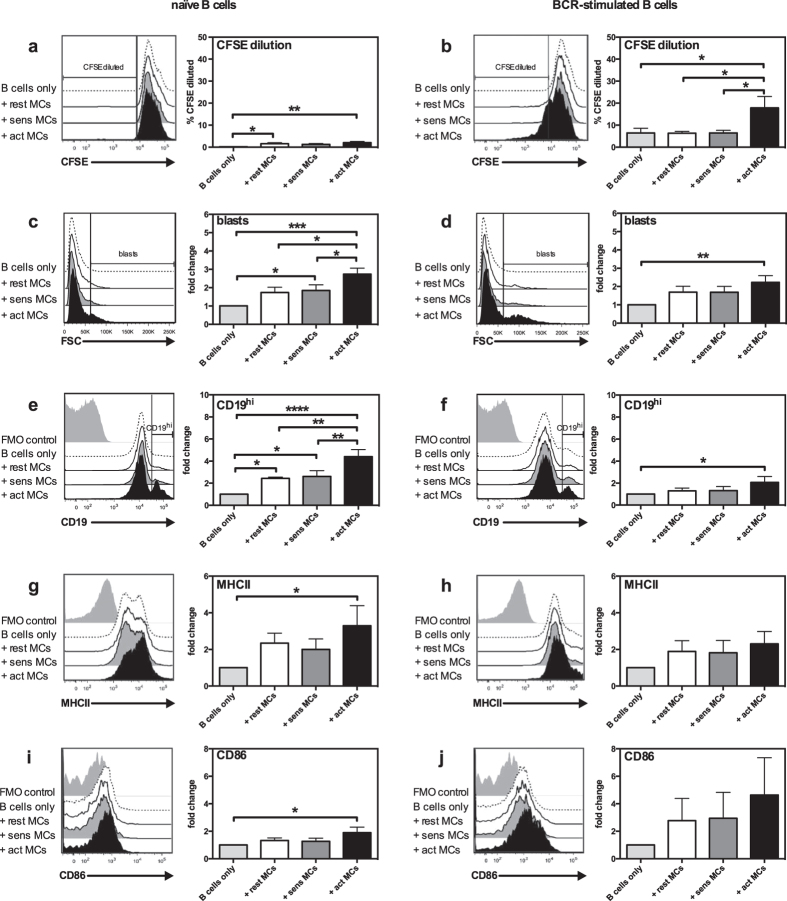
MCs promote proliferation and activation of naïve and BCR-activated B cells. Resting MCs (rest MCs), IgE-sensitized MCs (sens MCs) or antigen-activated MCs (act MCs) were cocultured with naïve **(a, c, e, g, i)** or BCR-activated **(b, d, f, h, j)** CFSE-labelled splenic B cells for 3 days. Cultures were analysed using flow cytometry and proliferation was assessed as % of B cells with lower CFSE staining intensity than naïve B cells cultured alone (% CFSE diluted), as demonstrated in the histograms by the vertical line (**a,b**). Blast formation was determined as % B cells large in FSC (indicated by the vertical line in the histograms in (**c,d**). The % CD19^hi^ B cells was determined as depicted in histograms in (**e,f**) and B cell expression of MHCII (**g,h**) and CD86 (**i,j**) as median fluorescence intensity. Results are expressed as mean % CFSE diluted (**a,b**) or mean fold-change (as compared to naïve or BCR-activated B cells cultured alone) (**c–j**) + SEM and represent 4 independent experiments (n = 5). *p < 0.05, **p < 0.01, ***p < 0.001. BCR, B-cell receptor; FMO, fluorescence-minus-one; FSC, forward scatter; MC, mast cell; MHCII, class II MHC.

To study the influence of MCs on B cell proliferation and activation, we set up a system where CFSE-labelled splenic B cells were cocultured with either resting, IgE-sensitized or antigen-activated MCs. Cells were harvested after 3 days of culture, and were then stained for B cell surface markers followed by flow cytometry analysis. Only viable B cells were included in the analysis (Supplementary Fig. S1 online). As seen in Fig. 1a, the presence of MCs promoted proliferation of naïve B cells, an effect that was independent of the activation state of the MCs. Additionally, activated MCs induced BCR-activated B cells to proliferate more than when cultured alone (Fig. 1b). In addition to assessing B cell proliferation, we also investigated several parameters of B cell activation, including blast formation and surface expression of CD19, MHCII and the costimulatory molecule CD86. After coculture with activated MCs, increased blast formation of both naïve (Fig. 1c) and BCR-stimulated (Fig. 1d) B cells was apparent, as compared to control cultures of B cells cultured in medium only. In contrast, less proliferation and blast formation was seen when B cells were cultured with non-activated MCs that were either resting (not sensitized) or IgE-sensitized only (without cross-linking antigen) (Fig. 1c,d), hence indicating that the expression/secretion of B cell blast-promoting factors by MCs is dependent on MC activation. Additionally, coculture with MCs resulted in upregulation of cell surface CD19 in both naïve and BCR-stimulated B cells, especially when the MCs were antigen-activated (Fig. 1 e,f). Moreover, there was an upregulation of cell surface MHCII in naïve B cells (Fig. 1g). BCR-activated B cells also showed a strong trend for increased MHCII expression after coculture with MCs, although this finding did not reach statistical significance (Fig. 1h). The upregulation of MHCII suggests that MCs can enhance the antigen-presenting capacity of B cells, a notion that was also supported by increased expression of surface CD86 (Fig. 1i,j). Notably, upregulated expression of MHCII and CD86 was only seen when the MCs had been subjected to IgE receptor cross-linking, suggesting that the ability of MCs to enhance B cell function is dependent on their activation state.

The data above suggest that MCs have profound effects on the activation status of B cells. However, it was important to evaluate whether, conversely, B cells might have a functional impact on MCs. To evaluate this possibility, we cocultured MCs with naïve or BCR-activated B cells followed by an assessment of MC activation markers. To monitor MC degranulation we measured β-hexosaminidase release, while late-phase MC activation was measured as upregulation of the IL-6 gene. As depicted in Supplementary Fig. S4 online, neither naïve nor BCR-stimulated B cells had any detectable effect on MC activation, confirming that the effects seen in the cocultures were due to antigen-activation of the MCs.

### The expression of L-selectin on cultured B cells is dependent on the presence of MCs

**Figure 2:**
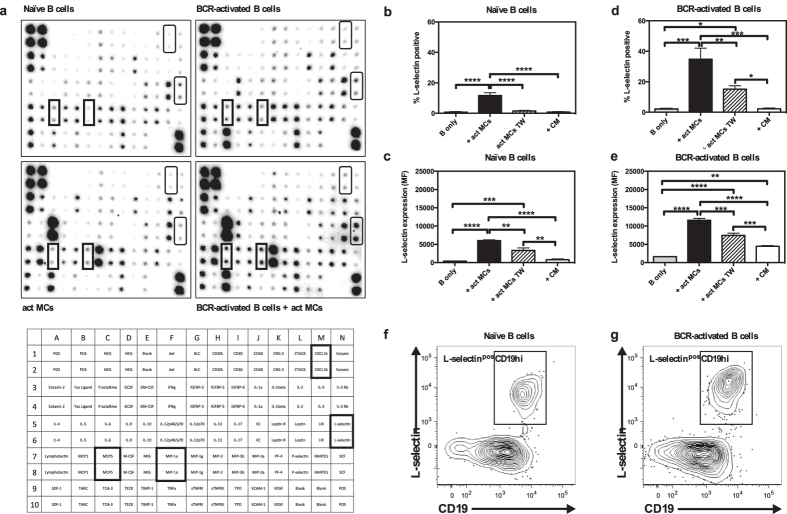
Activated MCs induce L-selectin expression on B cells. (**a**) Cytokine production in supernatants from cultures of naïve or BCR-activated B cells, antigen-activated MCs, or cocultures of the two latter was analysed using a protein array. Proteins upregulated in cocultures are indicated by boxes; see lower panel for their identification. Naïve (**b,c**) or BCR-activated (**d,e**) splenic B cells were cocultured for 3 days with antigen-activated MCs (act MCs), either in direct contact or separated by a transwell membrane system (TW), or cultured alone with conditioned medium (CM) from 3-days old cultures of antigen-activated MCs. The cultures were analysed by flow cytometry and the % L-selectin^pos^ cells and the median fluorescence intensity of L-selectin expression was determined. Results are expressed as mean + SEM (n = 3). (**f,g**) Representative flow cytometry plots of naïve (**f**) or BCR-activated (**g**) B cells after coculture with act MCs, demonstrating the L-selectin^pos^CD19^hi^ population. *p < 0.05, **p < 0.01, ***p < 0.001; ****p < 0.0001. BCR, B-cell receptor; MC, mast cell

In order to search for factors that could be involved in the crosstalk between MCs and B cells, we cocultured BCR-stimulated B cells with activated MCs, and used a protein array system to analyse the output of a panel of immunological mediators. By using this approach, we noted that MCs were the dominating source of several of the detected compounds, such as IL-6, IL-3 and MIP-1γ/CCL9, whereas B cells were the dominating source of others, such as IL-2 (Fig. 2a; see Supplementary Table S1 online). Moreover, we noted elevated levels of several pro-inflammatory compounds, including soluble L-selectin, CXCL16, MCP5 and MIP-1α, as a result of the coculture conditions in comparison with their levels in cultures of either naïve B cells, BCR-activated B cells or activated MCs only (Fig. 2a; see Supplementary Table S1 online). Out of these, the increase in soluble (shed) L-selectin was most prominent and we therefore chose to focus further on this finding. L-selectin has been shown to promote B-cell homing to lymphoid tissue and sites of inflammation^36^, and we thus hypothesized that MCs could regulate L-selectin expression and/or shedding in B cells, potentially influencing B cell function. To confirm and extend the finding from the protein array approach, we used flow cytometry to analyse the expression of L-selectin on B cells cultured either alone or in the presence of activated MCs. Moreover, we asked whether the effects of MCs on L-selectin expression in B cells are dependent on cell-cell contact, as evaluated by using a transwell culture system that separates B cells and MCs by a membrane but allows cellular communication through soluble factors. In addition, we assessed the effect of conditioned medium from activated MCs, to account for the effects of soluble factors secreted by MCs but excluding effects of soluble factors whose secretion is dependent on crosstalk between the two cell types.

As shown in Fig. 2b, very few naïve B cells cultured alone expressed L-selectin, while a significant number of L-selectin^pos^ B cells were observed in cocultures with activated MCs. The L-selectin^pos^ B cells in the cocultures expressed higher amounts of the protein when compared to the rare L-selectin^pos^ B cells in the control cultures (Fig. 2c). Furthermore, the MC-driven increase in the percentage of L-selectin^pos^ B cells was dependent on cell-cell contact as very few L-selectin^pos^ B cells were found when using the transwell system (Fig. 2b). However, the L-selectin expression on naïve B cells was increased in the transwell system, suggesting that soluble factors secreted from activated MCs can cause partial upregulation of L-selectin (Fig. 2c). Addition of MC-conditioned medium alone to naïve B cells did neither affect the number of L-selectin^pos^ naïve B cells nor the expression intensity (Fig. 2b,c). BCR-activated B cells were more susceptible to MC-dependent upregulation of L-selectin than were the naïve B cells, both in terms of the number of L-selectin^pos^ B cells and the intensity of expression (Fig. 2d,e). Moreover, partial upregulation of L-selectin could be seen after addition of MC-conditioned medium to BCR-activated B cells (Fig. 2e), although MC-conditioned medium did not affect the percentage of L-selectin^pos^ BCR-activated B cells (Fig. 2d,e). Interestingly, as demonstrated in Fig. 2f,g, the L-selectin^pos^ B cells corresponded to the CD19^high^ expressing B cell population.

### MC-dependent B cell activation is partially dependent on cell-cell contact

**Figure 3:**
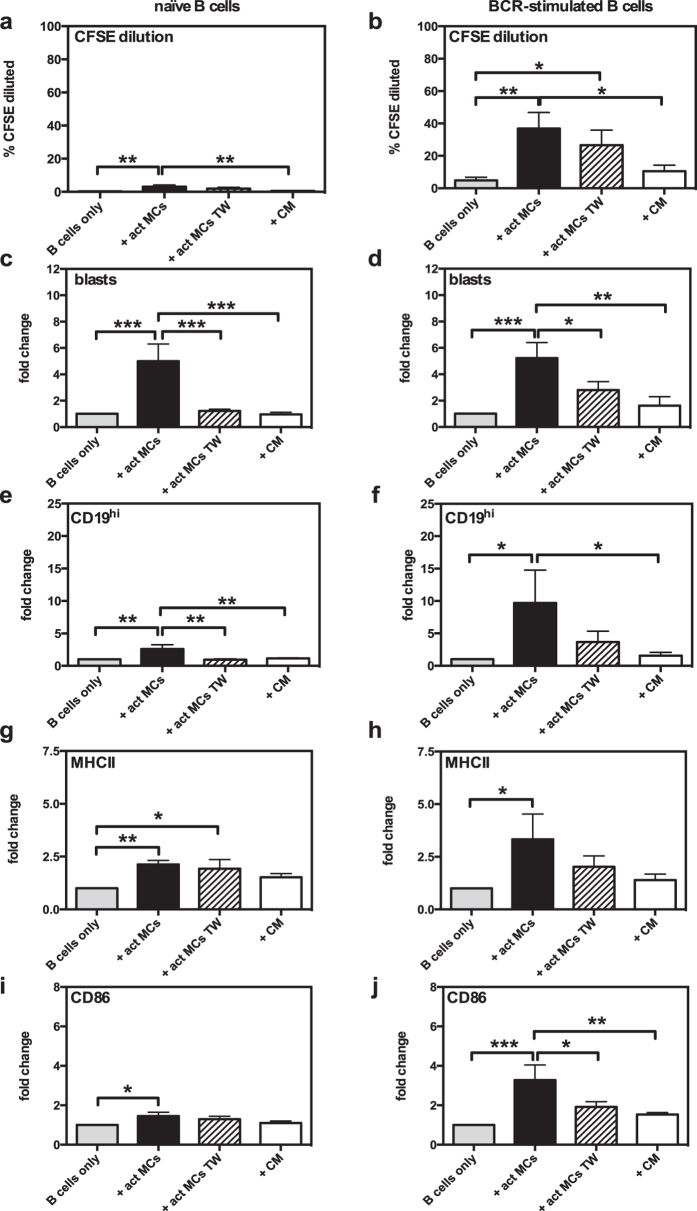
MC-dependent B-cell activation is partially dependent on cell-cell contact. Antigen-activated MCs (act MCs) were cocultured for 3 days with naïve (**a,c,e,g,i**) or BCR-activated (**b,d,f,h,j**) CFSE-labelled splenic B cells, separated or not by a transwell (TW) membrane system. Additional cultures were set up with naïve or BCR-activated CFSE-labelled splenic B cells cultured with conditioned medium (CM) from 3-days old cultures of antigen-activated MCs. Cultures were analysed using flow cytometry and proliferation was assessed as % of B cells with lower CFSE staining intensity than naïve B cells cultured alone (% CFSE diluted; (**a,b**). Blast formation was determined as % B cells large in forward scatter (**c,d**), the upregulation of CD19 was expressed as % of total B cells that are CD19^hi^ (**e,f**), and B-cell expression of MHCII (**g,h**) and CD86 (**i,j**) as median fluorescence intensity. Results are expressed as mean % CFSE diluted (**a,b**) or mean fold-change (as compared to naïve or BCR-activated B cells cultured alone) (**c–j**) + SEM and represent 3 independent experiments (n = 6). *p < 0.05, **p < 0.01, ***p < 0.001. BCR, B-cell receptor; MC, mast cell; MHCII, class II MHC.

While our data show that upregulation of L-selectin is, at least partially, dependent on cell-cell contact, previous studies show some discrepancies regarding the necessity for cell-cell contact in MC-dependent B-cell responses^14^,^37^. Next, we therefore examined whether, in our hands, the effect of MCs on the proliferation, blast formation and expression of B cell activation markers were dependent on cell-cell contact. To this end we again used the transwell system. In agreement with our observed dependence on cell-cell contact for induction of L-selectin, MC-driven increase in proliferation of naïve B cells was dependent on cell-cell contact (Fig. 3a). For BCR-activated B cells, increased proliferation was seen both in cocultures with cell-cell contact and in the transwell system, but not in cultures where MC-conditioned medium had been added to the B cells (Fig. 3b). This indicates that MCs and B cells are able to communicate without the need for direct cell-cell contact, such that soluble factors that drive B cell proliferation are produced. B cell blast formation was strongly dependent on cell-cell contact, both for naïve and BCR-activated B cells (Fig. 3c,d).

To further evaluate the mechanism by which MCs enhance the antigen-presenting potential of B cells, the dependence on cell-cell contact for expression of CD19, MHCII and CD86 was analysed. As seen in Fig. 3e,f, the increase in CD19 was dependent on cell-cell contact, both in cultures of naïve and BCR-activated B cells. The MHCII induction in naïve B cells was similar in direct vs. transwell cocultures, suggesting that direct cell-cell contact is not needed (Fig. 3g). However, there was no significant induction of MHCII when MC-conditioned medium had been added to B cells. Again, this suggests that communication between MCs and B cells through soluble factors may be sufficient to induce MHCII upregulation. In contrast, MHCII induction in BCR-activated B cells was clearly reduced both in transwell cultures and when MC-conditioned medium was administered (as opposed to direct cocultures), suggesting that cell-cell contact is needed for induction of MHCII in this B cell population (Fig. 3h). Similarly, the MC-driven upregulation of CD86 was dependent on cell-cell contact, both in naïve (Fig. 3i) and BCR-activated (Fig. 3j) B cells.

### MCs induce secretion of IgM and IgG by B cells

**Figure 4:**
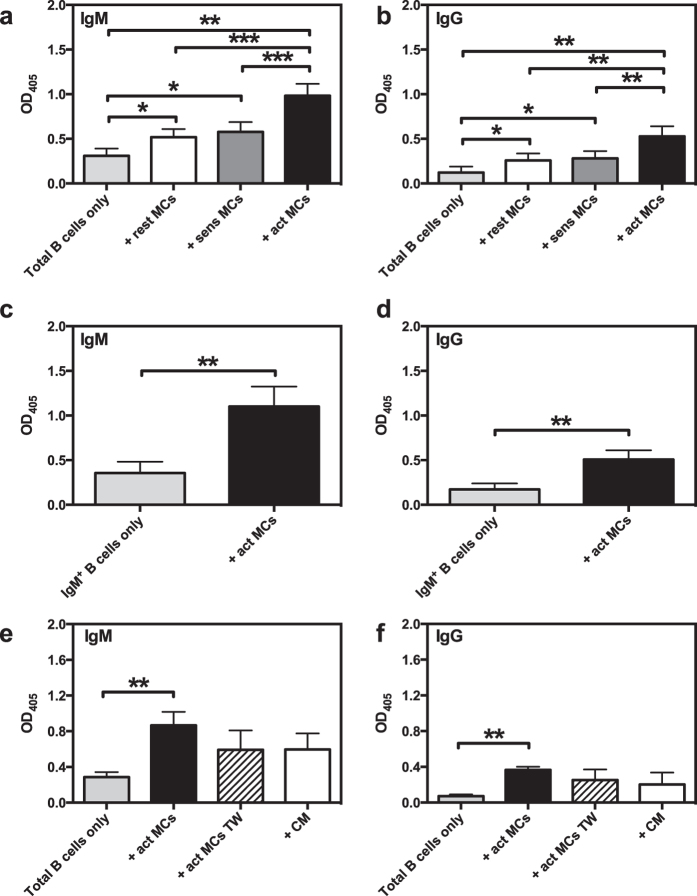
MCs induce B cells to secrete IgM and IgG. (**a,b**) Resting MCs (rest MCs), IgE-sensitized MCs (sens MCs) or antigen-activated MCs (act MCs) were cocultured with naïve splenic B cells. After 3 days, the culture supernatants were analysed by ELISA for secreted IgM (**a**) or IgG (**b**). Results are expressed as mean absorbance values at 405 nm + SEM and represent 4 independent experiments (n = 5). (**c,d**) Naïve splenic IgM^+^ B cells were FACS-sorted before cultured alone or with act MCs for three days. The culture supernatant were analysed by ELISA for secreted IgM (**c**) and IgG (**d**). Results are expressed as mean absorbance values at 405 nm + SEM and represent 2 independent experiments (n = 6). (**e,f**) Naïve splenic B cells were cultured together with antigen-activated MCs, separated or not by a transwell membrane system (TW). Additional cultures were set up with naïve splenic B cells cultured with conditioned medium (CM) from 3-days old cultures of act MCs. After 3 days the culture supernatants were analysed by ELISA for secreted IgM (**e**) or IgG (**f**). Results are expressed as mean absorbance values at 405 nm + SEM and represent 3 (**e**) or 1 (**f**) independent experiments (n = 3–5). *p < 0.05, **p < 0.01, ***p < 0.001, ****p < 0.0001. OD, optical density.

To further assess the ability of MCs to influence functional properties of B cells, we asked whether MCs could affect the production of antibodies by B cells. To this end, we analysed the supernatants from naïve B cell:MC cocultures for levels of secreted IgM and IgG using ELISA. As seen in Fig. 4a,b, there was a significant increase in antibody secretion when B cells were cocultured with MCs. Notably, antigen-activated MCs promoted a markedly higher increase in antibody production as compared with non-activated (resting or only sensitized) MCs. This was seen for both IgM and IgG, suggesting that MC-dependent B cell activation not only induces antibody secretion, but also promotes isotype class switching. To exclude that the secreted IgG was derived from IgG^+^ clones being present at the start of the culture, we also performed the experiment with FACS-sorted IgM^+^ B cells. This experiment confirmed that a class switch does indeed take place, as there was a substantial MC-dependent secretion of IgG also in these cultures (Fig. 4c,d). Additionally, our data show that antibody secretion was dependent on cell-cell contact, although there was a strong trend for both IgM and IgG secretion in cultures in the transwell system as well as when MC-conditioned medium was added (Fig. 4e,f).

### MCs induce activation of both MZ and FO B cells

**Figure 5:**
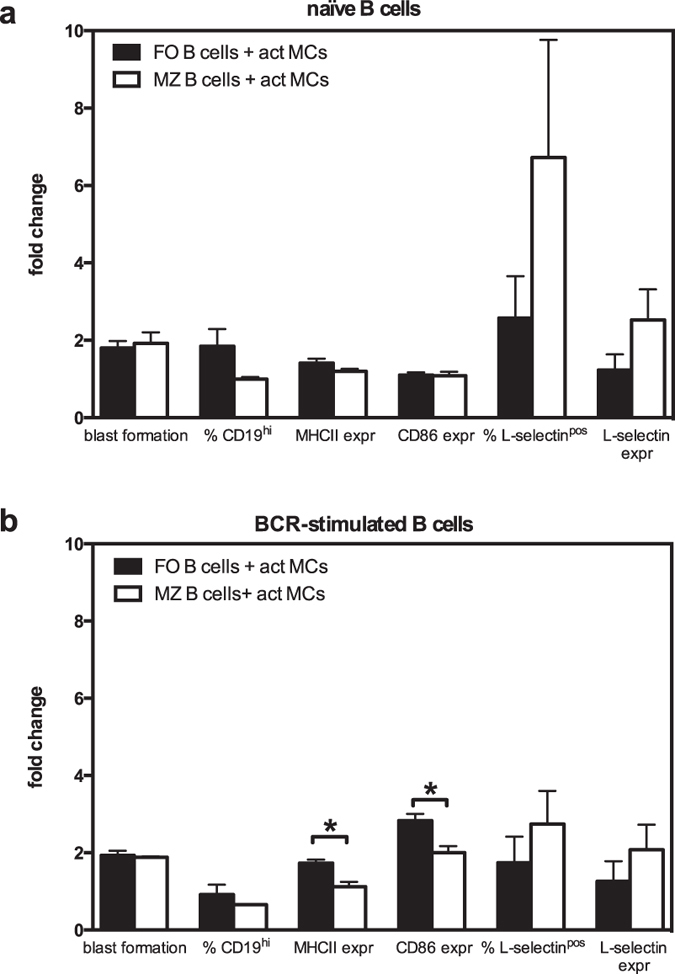
MCs influence different B-cell subsets. Splenic B cells were separated into marginal zone (MZ) and follicular (FO) B cells by FACS and were cultured alone or together with antigen-activated MCs (act MCs). The B cells were either naïve (**a**) or BCR-stimulated (**b**). The cultures were analysed by flow cytometry where blast formation was determined as % B cells large in FSC, the upregulation of CD19 was expressed as % CD19^hi^ of total B cells, and B-cell expression of MHCII and CD86 as median fluorescence intensity. The percentage of B cells positive for L-selectin (% L-selectin^pos^) and the L-selectin expression (median fluorescence intensity) was also determined. Results are expressed as mean fold change (as compared to naïve or BCR-stimulated B cell subset cultured alone) + SEM (n = 3). *p < 0.05. BCR, B-cell receptor; FSC, forward scatter; MHCII, class II MHC.

**Figure 6:**
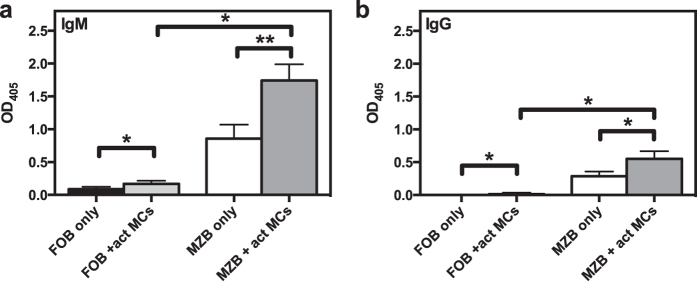
MCs have a larger effect on antibody secretion from MZ B cells than from FO B cells. Antigen-activated MCs (act MCs) were cocultured with naïve splenic marginal zone (MZ) or follicular (FO) B cells. After 3 days, the culture supernatants were analysed by ELISA for secreted IgM (**a**) or IgG (**b**). Results are expressed as mean absorbance values at 405 nm + SEM and are derived from 1 experiment (n = 3). *p < 0.05, **p < 0.01. OD, optical density.

To provide further insight into the influence of MCs on B cell function, we investigated the impact of MCs on FO and MZ B cells as these subsets have different immune functions. Accordingly, we cocultured FACS-sorted splenic MZ or FO B cells with activated MCs. When comparing the effect of MCs on these two B cell subsets, we detected no significant differences in terms of either blast formation or expression of CD19, MHCII or CD86 when naïve MZ or FO B cells were used (Fig. 5a). However, we noted that MZ B cells tended to show a greater increase than FO B cells in the proportion of L-selectin^pos^ cells and in the expression level of L-selectin on those (Fig. 5a). When examining the effect of activated MCs on BCR-stimulated MZ and FO B cells, we found that FO B cells were more sensitive than MZ B cells to MC-driven upregulation of MHCII and CD86 (Fig. 5b). Moreover, the trend of MC-dependent upregulation of L-selectin expression was less prominent in BCR-stimulated MZ B cells in comparison with naïve counterparts (Fig. 5b). We also analysed the culture supernatants for secreted IgM and IgG, and found that activated MCs could induce both MZ and FO B cells to secrete IgM as well as IgG (Fig. 6a,b). Notably, despite the smaller effect on activation markers compared to FO B cells, the MZ B cells showed larger MC-dependent fold increase in both IgM- and IgG-production.

## Discussion

It is now well established that MCs can contribute to a wide panel immunological reactions, of both innate and adaptive nature. In many cases, MCs impact on such conditions by influencing other immune cell populations. MCs are known to promote the recruitment of neutrophils^38^ and eosinophils^39^ and to promote the migration of dendritic cells to lymph nodes^40^. It is also well documented that MCs can interact with various types of T cells^41^,^42^,^43^,^44^,^45^,^46^,^47^ by inducing cytokine production through a mechanism dependent on MC-derived TNF^43^,^44^ and by promoting Treg and CD8^+^ T cell responses^42^,^47^. Conversely, there are also several studies indicating that T cells can cause activation of MCs^41^,^45^,^46^.

Given that MCs are implicated in various B cell-driven diseases, including arthritis, it is likely that MCs may also have an impact on these cells. Already in the sixties it was demonstrated that MCs accumulate within a few hours in the draining lymph nodes of mice upon subcutaneous antigen exposure^48^, suggesting that MCs participate in the mechanisms of antibody synthesis. It is also well known that B cell follicles can be established in arthritic joints^49^,^50^ and, given that MCs are recruited to such sites, it appears feasible that MCs can impact on these. However, the functional consequences of MC:B cell interactions have hitherto only been scarcely studied. In one early study, Mecheri and co-workers established that MCs can interact with B cells, and it was also shown that B cells were activated in terms of proliferation and blast formation when cocultured with MCs^37^. More recently, Pucillo and colleagues have confirmed the ability of MCs to induce B cell proliferation and blast formation^14^. Moreover, it was demonstrated that MCs could induce B cells to produce IL-10 and IgA, hence suggesting that MCs promote an anti-inflammatory phenotype of B cells^14^,^15^.

In this study we have expanded the functional consequences of MC:B cell interactions, with one important aspect being to evaluate whether the activation status of MCs has an impact on their influence over B cell populations. This has not been thoroughly investigated in previous studies on this topic, where MCs have been mostly evaluated at either base line conditions, i.e. without previous activation, or after crosslinking of FcεRs.

In some contrast to earlier studies, our data point to a role for MCs in promoting effector parameters of B cells, as opposed to earlier studies indicating that MCs promote an anti-inflammatory phenotype of B cells^14^,^15^. In agreement with previous studies^14^,^37^, we show that MCs have the capacity to induce proliferation and blast formation of both naïve and BCR-activated B cells. Moreover, we assessed whether the activation status of MCs could have an impact on the response of B cells and, indeed, our data indicate that MC activation has a profound enhancing effect on the extent of B cell response in terms of proliferation and blast formation. The upregulation of CD19 on B cells in the cocultures demonstrates that MCs can lower the activation threshold in B cells. Thus, an elevated expression of CD19 will render the B cells hyper-responsive to antigen and innate signals, a state that is associated with production of autoantibodies^51^,^52^,^53^,^54^. As additional signs of B cell activation driven by MCs, we showed that coculture of MCs with B cells resulted in a marked upregulation of MHCII and of CD86, suggesting that MCs can enhance the antigen-presenting capacity of B cells. In agreement with our observed effects on B cell proliferation and blast formation, robust upregulation of MHCII and CD86 was only seen when MCs had been activated to degranulate. In this context, it is important to emphasize that MCs often show signs of activation in pathological situations; for example, MCs are frequently degranulated in arthritic joints^17^. Our data thus suggest that one of the consequences of MC activation/degranulation is to promote an optimal B cell response.

We also demonstrate that activated MCs support the expression of L-selectin on B cells. Intriguingly, we found that the level of soluble (shed) L-selectin was enhanced when MCs were co-cultured with B cells, introducing the possibility that MCs promote L-selectin shedding from the B cell surface. However, flow cytometry analysis revealed that also the cell surface expression of L-selectin was enhanced on the B cells that had been co-cultured with MCs. Thus, a likely explanation for the increase in soluble L-selectin in B cell:MC co-cultures may lie within the overall stimulation of L-selectin expression in B cells, as reflected both by increased cell surface levels and increased levels of shed L-selectin. L-selectin has been shown to be crucial for the homing of various leukocytes, including T cells and B cells, to lymph nodes and inflammatory sites^36^. L-selectin is rapidly shed from the cell surface following activation, and it has been suggested that this may represent a mechanism by which lymphocyte trafficking could be modulated, rapidly leading to accumulation of cells in an appropriate tissue^55^. The results presented here thus suggest that MCs can enhance the capacity of B cells for homing to such sites, which could be a mechanism to enhance the adaptive immune response to a given challenge. In addition, the MC-mediated up-regulation of L-selectin on the B cell surface could represent a mechanism that acts locally at sites of MC activation in the tissue, which could serve to sustain B cells at an inflammatory site, e.g. in ectopic lymphoid follicles in rheumatic joints.

In further agreement with an impact of MCs on B cell effector properties, we demonstrate that MCs promote the secretion of IgM by B cells, a finding that is in agreement with previous observations^37^. In addition, we show that MCs induce the secretion of IgG by B cells. Importantly, the enhancement of Ig production, both IgM and IgG, was strongly enhanced when MCs had been subjected to IgE receptor cross-linking, hence reinforcing the need for MC activation in inducing an optimal B cell response.

At present we cannot with certainty define the exact mechanism by which MCs induce B cell activation. Likely candidates having this effect include various cytokines, e.g. IL-4 and IL-6, which are known to promote Ig production in B cells. However, in an earlier study it was shown that inhibition of MC-derived IL-6 or IL-4 did not affect the amplitude of B cell responses, arguing against this possibility^37^. On the other hand, these findings were challenged in a more recent study where evidence was presented that supported a role for MC-derived IL-6 in promoting B cell proliferation^14^. Indeed, considering the remarkable production of IL-6 by MCs, as seen both in this and other studies, it appears likely that MC-derived IL-6 may have an impact on B cells. However, the exact mechanism by which MCs induce B cell activation remains to be established.

Earlier studies have to some extent investigated whether the impact of MCs on B cells is dependent on cell-cell contact. In one study, it was suggested that cell-cell contact is dispensable for inducing B cell proliferation and blast formation as shown by the ability of MC-conditioned medium to produce such responses^37^. In contrast, another study indicated that cell-cell contact was partly needed for optimal MC-driven stimulation of B cell proliferation^14^. In our hands, cell-cell contact was required for MCs to induce B cell blast formation and upregulation of CD19, whereas there was only a trend of cell-cell contact dependency for MC-driven induction of B cell proliferation. Moreover, our data indicate that the induction of CD86 is partly dependent on cell-cell contact, whereas there was no clear dependency of cell-cell contact for the upregulation of MHCII on naïve B cells. Together, our findings thus suggest that MCs promote B cell activation by a combination of cell-cell contact-dependent and -independent mechanisms. As regards the nature of the cell-cell contacts between MCs and B cells, previous findings have suggested that CD40-CD40L interactions may contribute^14^.

An important finding in this investigation was that MCs promote the production of IgG in B cells. 

Intriguingly, the induction of IgG secretion suggests that MCs promote CSR in B cells, a process that typically requires cell-cell contact between the B cells and T cells^56^,^57^. However, there was a strong trend of IgG secretion also in the transwell system and when B cells were cultured with MC-conditioned medium, thus suggesting that MCs can, albeit less efficiently, also induce B cell CSR by a route that bypasses the need for cell-cell contact. Although we cannot define the exact mechanism behind this, one explanation could be CD40L-expressing exosomes secreted by the activated MCs^15^,^58^. It is also notable that recent reports have shown that certain soluble factors, such as APRIL and BAFF^5^,^59^, can induce B cell CSR without need for direct cell-cell contact. Potentially, activated MCs may thus have the capacity to secrete such CSR-inducing soluble factors. According to such a scenario, it is conceivable that our observed higher sensitivity of MZ vs. FO B cells to Ig production in response to soluble factors released by MCs could be explained by the reported higher sensitivity of MZ vs. FO B cells to soluble CSR-inducing factors such as BAFF^60^.

In conclusion, we have demonstrated that MCs can enhance the effector cell properties of B cells in an *in vitro* setting. Future analyses are warranted to evaluate the *in vivo* significance of the present findings.

## Additional Information

**How to cite this article**: Palm, A.-K. E. *et al*. Activated mast cells promote differentiation of B cells into effector cells. *Sci. Rep*. **6**, 20531; doi: 10.1038/srep20531 (2016).

## Supplementary Material

Supplementary Information
