# Supplementary material for: Activated mast cells promote differentiation of B cells into effector cells
Source: Sci Rep. 2016 Feb 5;6:20531. doi: 10.1038/srep20531 (PMC4742803; doi:10.1038/srep20531)
Supplement: Supplementary Information [file srep20531-s1.pdf]

Supplementary information

Activated mast cells promote differentiation of B cells into effector cells

Anna-Karin E. Palm[1], Gianni Garcia Faroldi[2], Marcus Lundberg[1], Gunnar Pejler[3,2] and Sandra Kleinau[1*]

[1] Uppsala University, Department of Cell and Molecular Biology, Uppsala, Sweden

[2] Swedish University of Agricultural Sciences, Department of Anatomy, Physiology and Biochemistry, Uppsala, Sweden

[3] Uppsala University, Department of Medical Biochemistry and Microbiology, Uppsala, Sweden

Supplementary Table S1. Quantification of the cytokine array presented in Fig. 2a. Values are mean expression intensity ± standard deviation of duplicates for every spot on the cytokine array, as quantified using the ImageJ software. Mediators indicated in bold represent those that are profoundly up-regulated in co-cultures of MCs and B cells vs. their expression in cultures of MCs or B cells alone.

Mediator	Naïve BCs	BCR-act. BCs	Act. MCs	BCR-act. BCs + act. MCs
AXL	2.38±0.69	3.10±0.68	1.35±0.65	2.49±0.07
BLC	14.10±1.36	14.83±1.69	8.85±0.89	14.42±3.03
CD30L	3.62±0.48	9.85±0.05	1.63±0.74	6.00±0.10
CD30	3.02±0.49	4.67±1.48	2.10±0.36	3.89±0.92
CD40	0.81±0.03	1.69±0.42	0.45±0.09	2.00±1.09
CRG-2	0.87±0.42	6.84±0.39	1.03±0.06	6.70±2.12
CTACK	6.49±0.77	10.06±1.96	6.47±1.05	13.54±6.96
**CXCL16**	**2.34±0.61**	**6.13±1.09**	**3.18±0.48**	**12.79±0.28**
Eotaxin	5.10±0.28	8.69±0.86	4.45±0.23	5.69±1.64
Eotaxin-2	100.68±1.45	100.61±0.12	117.39±5.44	121.79±9.19
Fas Ligand	33.41±7.60	39.93±1.11	33.42±0.63	40.15±6.04
Fractalkine	4.46±0.56	4.78±0.68	6.29±3.32	6.36±2.67
G-CSF	2.46±0.16	3.22±0.46	2.15±0.64	4.59±2.05
GM-CSF	1.82±0.86	1.93±0.54	29.48±8.24	25.87±0.01
IFNγ	1.34±0.23	4.65±0.27	1.72±0.54	4.49±0.45
IGFBP-3	15.13±1.45	17.38±0.47	10.89±0.52	17.77±5.26
IGFBP-5	12.34±2.87	15.51±0.66	11.21±0.60	20.85±0.39
IGFBP-6	2.90±0.01	3.79±0.15	1.52±0.10	3.29±0.57
IL-1α	24.56±2.16	35.38±0.62	22.19±2.69	34.32±0.80
IL-1β	3.61±0.30	6.12±0.60	2.95±0.00	11.86±3.98
IL-2	2.80±0.38	35.11±0.40	3.44±0.09	34.71±5.80
IL-3	1.55±0.00	5.44±0.38	35.58±1.44	37.20±5.20
IL-3β	6.02±0.23	9.24±0.71	13.39±1.15	20.77±3.20
IL-4	12.68±2.37	12.29±2.32	11.41±2.63	14.09±3.39
IL-5	2.61±0.40	5.60±0.29	7.36±0.32	13.74±1.65
IL-6	1.28±0.09	1.98±0.52	199.61±3.81	210.45±1.01
IL-9	10.15±1.52	42.39±3.87	11.19±0.32	38.95±2.75
IL-10	5.25±0.39	6.84±0.85	0.81±0.24	6.26±0.01
IL-12p40/p70	10.44±0.93	12.98±3.31	2.92±1.20	11.63±0.30
IL-12p70	12.45±0.45	17.75±4.01	10.05±0.54	15.68±1.67
IL-13	3.64±0.43	7.22±1.57	8.25±1.40	12.24±2.05
IL-17	0.40±0.16	1.43±0.30	0.19±0.13	0.56±0.11
KC	2.60±0.85	4.04±0.97	1.26±0.30	3.98±0.40
LeptinR	19.15±1.67	25.42±4.66	16.64±2.42	24.57±0.06
Leptin	9.31±1.16	17.77±3.02	7.34±0.98	23.15±9.49
LIX	55.06±4.28	58.35±9.12	54.90±6.43	64.59±10.20
**L-Selectin**	**17.03±2.78**	**20.10±6.94**	**8.54±2.75**	**52.03±1.76**
Lymphotactin	51.78±1.19	56.21±0.97	50.84±7.60	58.33±0.53
MCP1	23.42±1.67	29.18±2.06	87.62±5.43	103.32±2.95

**MCP5**	**12.68±1.37**	**19.02±2.59**	**15.28±0.12**	**36.22±2.98**
M-CSF	25.48±0.24	30.99±0.49	21.33±4.94	31.95±3.18
MIG	32.20±0.16	31.71±1.64	26.78±0.29	34.32±0.42
**MIP-1α**	**4.69±1.61**	**15.11±0.67**	**21.14±1.03**	**41.33±2.43**
MIP-1γ	24.56±1.54	54.13±3.90	128.16±1.38	162.76±5.50
MIP-2	34.51±1.53	42.37±2.45	37.38±1.25	48.62±1.28
MIP-3β	45.78±0.67	54.03±1.92	43.64±0.92	53.59±3.89
MIP-3α	9.39±0.80	15.63±2.55	6.57±0.18	13.00±0.01
PF-4	21.91±1.95	25.70±1.03	16.20±1.50	20.34±6.06
P-Selectin	29.39±0.61	35.17±2.37	26.17±1.39	32.22±6.82
RANTES	13.46±0.77	15.64±1.90	13.27±4.49	22.92±13.29
SCF	4.46±1.25	6.45±0.16	3.37±0.81	15.39±6.81
SDF-1	62.06±1.15	65.68±0.93	49.38±4.17	59.15±5.88
TARC	6.47±0.73	17.78±1.58	4.83±0.24	16.02±2.65
TCA-3	22.74±0.19	127.42±0.59	80.09±5.16	143.16±2.97
TECK	3.92±0.42	11.03±1.59	2.03±0.84	13.38±0.78
TIMP-1	14.93±1.93	20.07±1.24	9.11±2.62	23.87±0.35
TNFα	4.14±0.01	5.73±1.21	1.65±0.03	5.36±0.67
sTNFRI	2.35±0.39	3.79±0.06	2.91±0.55	11.38±1.10
sTNFRII	7.89±0.21	11.92±2.99	6.15±0.26	24.85±0.37
TPO	32.63±1.95	71.60±4.41	27.21±0.34	56.34±4.04
VCAM-1	6.20±0.20	10.10±1.46	1.03±0.35	19.44±10.51
VEGF	10.33±1.78	17.73±4.47	8.58±1.61	23.46±6.34

Supplementary figure S1

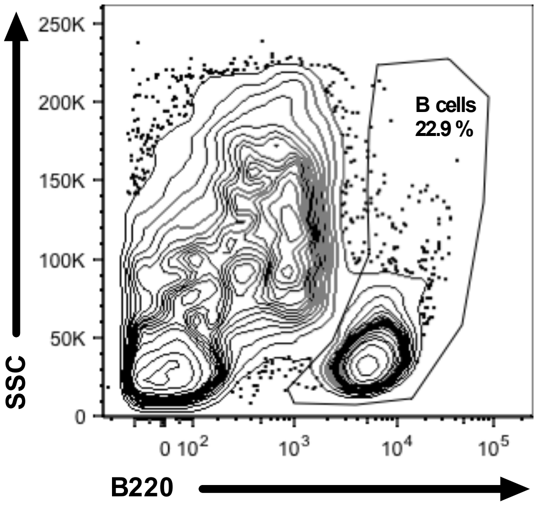

Supplementary Figure S1. Gating strategy for B cells. Cells from the MC:B cell cocultures were stained for surface markers and the viability dye 7-AAD and analyzed by flow cytometry. B cells were defined as 7-AAD$^-$ B220^{high}. SSC = side scatter

Supplementary figure S2

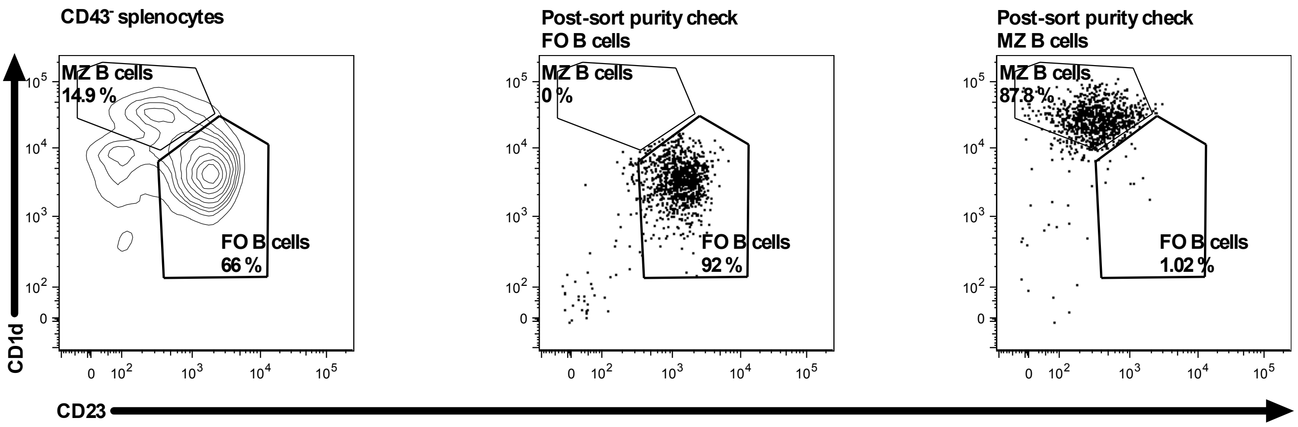

Supplementary Figure S2. Sorting strategy for MZ and FO B cells. Splenocytes were first enriched for B cells using MACS, and subsequently stained for CD1d and CD23 and sorted into marginal zone (MZ) and follicular (FO) B cells using FACS. MZ B cells were defined as CD1d^{high} CD23^{low} and FO B cells as CD1d^{low} CD23^{high} (left). Sorting purities were routinely around 90% for both FO B cells (middle) and MZ B cells (right). FO B cells, follicular B cells; MZ B cells, marginal zone B cells.

Supplementary figure S3

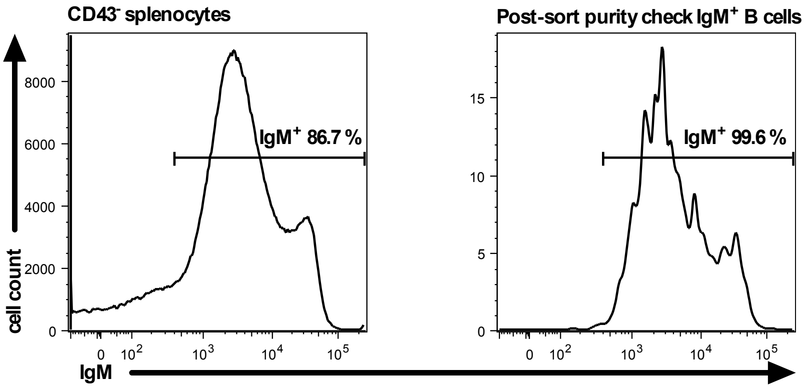

Supplementary Figure S3. Sorting strategy for IgM$^+$ B cells. Splenocytes were first enriched for B cells using MACS, and subsequently stained for IgM. The IgM$^+$ fraction was routinely sorted with a purity of >98%.

Supplementary figure S4

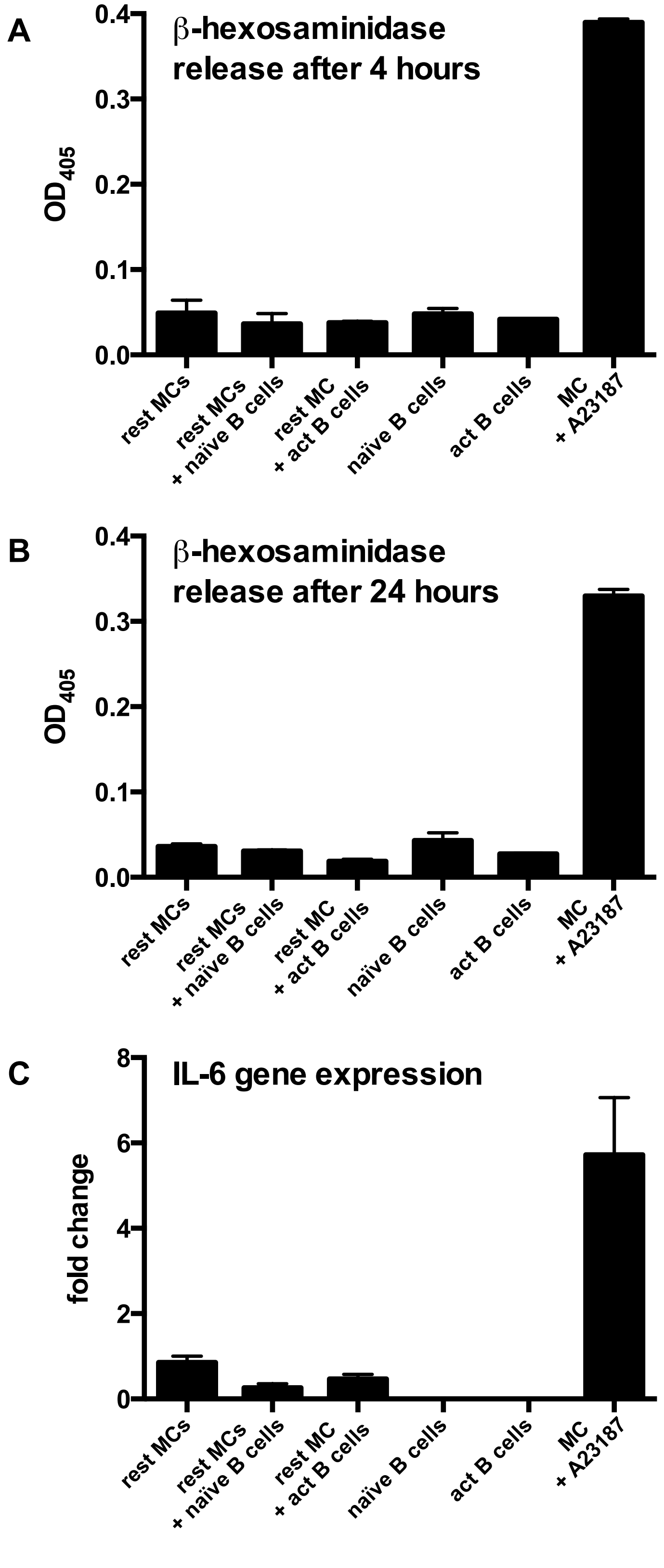

Supplementary Figure S4. Coculture of MCs and B cells does not provoke MC activation. Non-activated MCs (rest MCs) were cocultured with naïve or BCR-activated B cells (act B cells) and MC activation was monitored as the release of β-hexosaminidase (A, 4 hours incubation; and B, 24 hours incubation) and the expression of the IL-6 gene as determined by real time quantitative RT-PCR (C, 4 hours incubation). As negative controls, rest MCs, naïve or act B cells were cultured alone. As positive control for MC activation, MCs were incubated with 1 µM calcium ionophore A23187. Note that stimulation of MCs by calcium ionophore produces a robust β-hexosaminidase release and profound IL-6 gene upregulation, whereas coculture of MCs with B cells does not result in MC activation. Results are expressed as mean + SEM and represent 2 independent experiments in duplicates. MC, mast cell; BCR, B cell receptor
